# Royal Orthopædic Hospital

**Published:** 1903-07-25

**Authors:** 


### ROYAL ORTHOPÆDIC HOSPITAL.

Mr. H. A. Reeves writes from 70a Grosvenor Street, W., denying that a circular was sent out signed by several governors as a protest against the scheme of amalgamation. He further states that the alternative scheme for rebuilding was put to the meeting and voted upon. It was not ruled out of order, as our representative was informed.

